# Extended Kalman filter algorithm for non-roughness and moving damage identification

**DOI:** 10.1038/s41598-022-26339-z

**Published:** 2022-12-19

**Authors:** Hong-li Ding, Chun Zhang, Yu-wei Gao, Jin-peng Huang

**Affiliations:** 1 Civil Engineering and Architecture School, Nanchang University, Nanchang, People’s Republic of China; 2 Jiangxi Institute of Economic Administrators, Nanchang, People’s Republic of China

**Keywords:** Civil engineering, Applied mathematics

## Abstract

It is a promising method to identify structural damage using bridge dynamic response under moving vehicle excitation, but the lack of accurate information about road roughness and vehicle parameters will lead to the failure of this method. The paper proposed a step-by-step EKF damage identification method, which transforms the inversion problem of unknown structural parameters under unknown loads (vehicle and road roughness) into two separate inversion problems: moving contact force identification and damage parameters identification. Firstly, the VBI model is converted into bridge vibration model under a moving contact force, and the moving contact force covering the information of road roughness and vehicle parameters can be calculated by EKF iteration. Secondly, the moving contact force identified in the first step is loaded on the bridge as a known condition, and the bridge damage problem is also solved by the EKF method. Numerical analyses of a simply-supported bridge under the moving vehicle are conducted to investigate the accuracy and efficiency of the proposed method. Effects of the vehicle speed, the damage cases, the measurement noise, and the roughness levels on the accuracy of the identification results are investigated. The results demonstrate the proposed algorithm is efficient and robust, and the algorithm can be developed into an effective tool for structural health monitoring of bridges.

## Introduction

Bridge structures may suffer different degrees of damages due to environmental, long-term load effects and other factors. The accumulation of damage will reduce the service life of the bridge and even pose a huge threat to human life and property safety.

Among the commonly used detection methods, research on bridge damage identification using vehicle-bridge coupling vibration signals is paid more attention. Vehicle-induced vibration response is more significant than free vibration of a bridge, and the moving vehicle will excite each point of the bridge in turn to amplify the effects of local bridge damages. On the other hand, using moving vehicles as incentives makes it possible to not interrupt traffic during structural damage identification.

The damage identification method based on vehicle-bridge coupling vibration signals can be divided into: signal-based method and model-based method^1^. In the signal-based method, researchers analyzed the bridge dynamic responses under moving loads and identified structure damages by using signal-processing techniques such as wavelet analysis^2–6^, Hilbert Huang Transform (HHT)^7,8^ and so on. The features extracted from the vibration signals of the structure can be applied to determine damage occurrence and geometric location. Model-based methods and model updating techniques are preferred for further assessment of the damage. Structural damage leads to changes in structural modal parameters. Some researchers^9–11^ tried to establish the relation between the modal parameters and damages, and then structural modal parameters were extracted from bridge responses to detect local damage. More accurate damage identification results can be obtained by using bridge responses in time domain when a known moving load or vehicle was used as excitation^12–14^. Recently, Zhan et al.^15^ used train-induced responses and sensitivity-based FE model updating method to evaluate the damages of railway bridges. Gökdağ^16^ identified the crack information of beam-type structure under moving vehicle by minimizing the difference of the measured and the calculated responses in the particle swarm optimization. Feng et al.^17^ measured displacement response of a short-span railway bridge to freight trainloads using a low-cost remote vision sensor, and updated the railway bridge stiffness using time-domain optimization.

However, the moving vehicular parameters and the road surface roughness profile are generally hard to obtain accurately in practical engineering. Accurate identification of unknown vehicle-bridge interaction force is a key point in detecting bridge damages. In the research of the moving force identification (MFI) method, Law^18^ related the Fourier transforms of the moving forces and the responses in the frequency domain, and the time histories of the forces can be obtained by the least-squares method. In the time domain method (TDM)^19^, the forces were presented as step functions in a small time interval, and then the differential equations about structure and forces models can be solved by deconvolution in the time domain. Since the MFI is inherent ill-posed^20^, it was found that the identification results always suffer large fluctuations and sensitive to noise. To overcome the ill-posedness of MFI, some regularization techniques, such as Tikhonov regularization^21^, $l1$-norm regularization^22^ and truncated generalized singular value decomposition method (TGSVD)^23^, are employed to stabilize the computations and suppress the effects of noise^24^. Combining the MFI method, the iterative methods for sequentially identifying moving forces and bridge damages are proposed. Zhu and Law^25^ proposed a method to identify the time histories of moving forces and bridge damage with a two-step identification procedure iteratively. Later, the simultaneous identification of the time-histories of the interaction forces and system parameters is used to study the structural condition assessment problem in an iterative manner^26^.

In the above work on moving-load and damage identification, all response data of the vehicle passing through the bridge need to be used to form a large system matrix. High-dimensional system matrix will bring greater computational burden and stronger ill-posedness. Compared with the above-mentioned one-off methods, the recursive algorithm^27–30^ can use only the response data at the current time step to identify the structural parameters or loads in each recursive step, the damage parameters are continuously updated over time. As a typical recursive algorithm, the extended Kalman filter (EKF) method provides the minimum variance estimation of estimated variables with no need of accurate initial values of the motion state of the structure. Feng^27^ applied Bayesian inference regularization to identify bridge structural parameters and vehicle dynamic axle loads. Chen and Lee^28^ proposed an adaptive algorithm based on Kalman filter to identify moving loads without taking the road roughness into account. Considering temperature effects, Jin^29^ detected damage for a highway bridge with an extended Kalman filter-based artificial neural network (EKFNN) method. Zhang et al.^30^ used the EKF and $l1$-norm regularization technique to identify bridge damages under moving vehicular load.

There are two classic inverse problems in the bridge health monitoring, moving force identification^18^ and the damage identification^31^. Meanwhile, the ill-poseness of the inverse problem is serious and the results may not converge due to the unknown vehicle parameters and road roughness. Based on the EKF method with $l1$-norm regularization, this paper proposes a step-by-step identification method for vehicle-bridge force and bridge structure damages without information of the road roughness and moving vehicle.

Initially, the classic VBI model is transformed into a bridge vibration model under the time-varying excitation caused by the moving vehicles. The information of road roughness and vehicle parameters will be covered in the contact force time history. With the EKF method, two augmented state vectors of moving force and bridge damage parameters are deduced through the VBI model functions. Firstly, the moving force can be calculated by EKF iteration. Secondly, with the moving force identified in the first step, and the bridge damage problem is also solved by the EKF iteration.

With an example of a numerical analyses of a simply-supported bridge under the moving vehicle, iterative calculations can obtain the accurate results of the moving force and the bridge damages. It was demonstrated that the approach could successfully detect the damage in the presence of unknown road roughness and moving vehicle parameters.

## Basic theory

### Vehicle-bridge coupling vibration responses to identify bridge damage

The bridge response subjected to a moving vehicle is used to detect bridge damages, vehicle-bridge interaction system is the foundation of the model-based damage identification algorithm.

**Figure 1:**
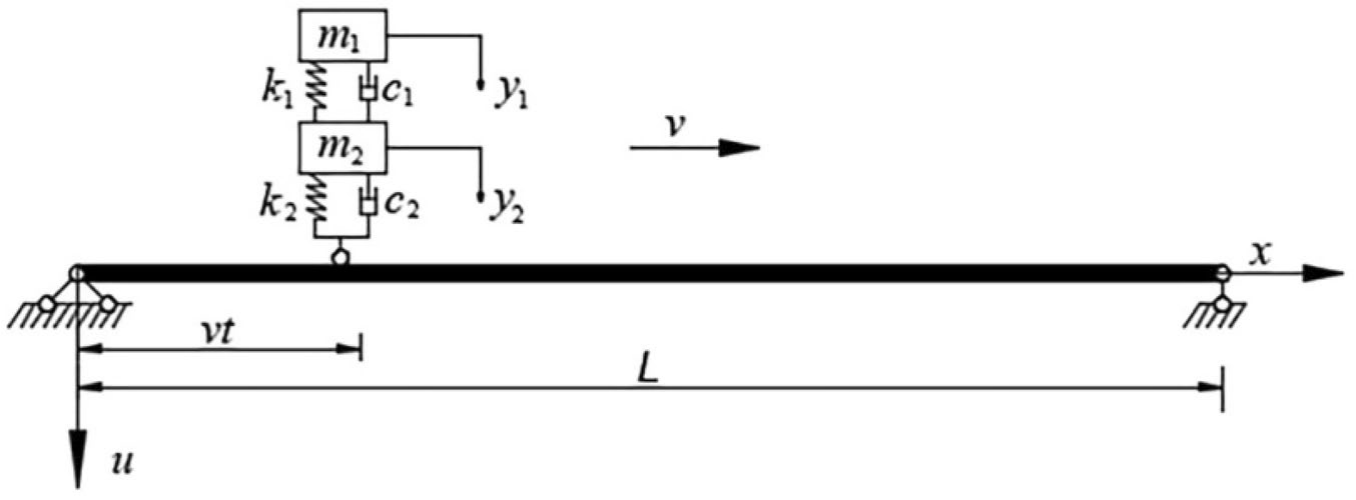
Beam subjected to a moving vehicle.

A continuous uniform Euler–Bernoulli beam bridge subject to a moving vehicle is shown in Fig. 1. The beam parameters include beam span $L$, Young’s modulus $E$, mass density $\rho$, cross-section area $A$, damping $c$, and moment of inertia $I$, and the parameters of the moving vehicle which is modeled as a oscillator are sprung mass ${m}_{1}$, spring stiffness ${k}_{1}$, damping coefficient ${c}_{1}$, and unspring mass ${m}_{2}$, unspring stiffness ${k}_{2}$, damping coefficient ${c}_{2}$. The vehicle is assumed to be moving at a prescribed velocity $v$ along the axial direction of the beam from left to right. The contact surface between the vehicle and the bridge is assumed to be rough and remain in contact with each other. With the assumptions mentioned above, the motion equations of the VBI model illustrated in Fig. 1 can be derived as:

1 $${m}_{1}\ddot{z}\left(t\right)+{k}_{1}[z\left(t\right)-u\left(x,t\right){|}_{x=vt}+{\left.r\left(x\right)\right|}_{x=vt}]+{c}_{1}\left[\dot{z}\left(t\right)-\frac{\partial u\left(x,t\right)}{\partial t}{|}_{x=vt}+{\left.\dot{r}\left(x\right)\right|}_{x=vt}\right]=0$$

2 $$m\frac{{\partial }^{2}u\left(x,t\right)}{\partial {t}^{2}}+c\frac{\partial u\left(x,t\right)}{\partial t}+\frac{{\partial }^{2}}{\partial {x}^{2}}\left[EI\left(x\right)\frac{{\partial }^{2}u\left(x,t\right)}{\partial {x}^{2}}\right]=\delta \left(x-vt\right)f\left(x,t\right)$$

where $x$ is the location in the longitudinal direction, $t$ is the time, $u\left(x,t\right)$ is the vertical displacement of the bridge, $z\left(t\right)$ is the displacement of ${m}_{1}$, $r\left(x\right)$ is the road roughness and $\delta$ is Dirac delta function.

In Eq. (2), the contact force $f\left(x=vt,t\right)$ between the bridge and the vehicle can be written as:

3 $$f = \left({m}_{1}+{m}_{2}\right)g-{m}_{2}{\left.\frac{{\partial }^{2}u\left(x,t\right)}{\partial {t}^{2}}\right|}_{x=vt}+{k}_{1}[z\left(t\right)-u\left(vt,t\right)+r\left(vt\right)]{+c}_{1}\left[\dot{z}\left(t\right)-{\left.\frac{\partial u\left(x,t\right)}{\partial t}\right|}_{x=vt}+\dot{r}\left(vt\right)\right]$$

The vibration displacement response of the bridge can be expressed as:

4 $$u\left(x,t\right)=\sum_{i=1}^{N}{p}_{i}\left(t\right){\varphi }_{i}\left(x\right)$$

where $N$ is the order of modal truncation, ${\varphi }_{i}\left(x\right)$ and ${p}_{i}\left(t\right)$ are *i*th mass normalized mode shape and *i*th general modal coordinate, respectively.

Based on the orthogonality conditions, substituting the Eqs. (3) and (4) into Eqs. (1) and (2), the governing equation can be represented as follows:

5 $${\varvec{M}}\ddot{{\varvec{q}}}\left(t\right)+{\varvec{C}}\dot{{\varvec{q}}}\left(t\right)+{\varvec{K}}{\varvec{q}}\left(t\right)={\varvec{F}}$$

where ${\varvec{q}}=[{{p}_{1}\dots {p}_{N }z]}^{T}$ is the generalized displacement vector, ${\varvec{M}}$, ${\varvec{C}}$, ${\varvec{K}}$ denote the generalized mass, damping, stiffness matrices of the bridge and ${\varvec{F}}$ is the generalized force vector, respectively.

6a $${\varvec{M}}=\left[\begin{array}{cc}{\varvec{I}}+{m}_{2}{\boldsymbol{\Phi }}^{T}\boldsymbol{\Phi }& {0}_{N\times 1}\\ {0}_{1\times N}& {m}_{1}\end{array}\right]$$

6b $${\varvec{C}}=\left[\begin{array}{cc}\boldsymbol{\Gamma }+{c}_{1}{\boldsymbol{\Phi }}^{T}\boldsymbol{\Phi }& -{c}_{1}{\boldsymbol{\Phi }}^{T}\\ {c}_{1}\boldsymbol{\Phi }& {c}_{1}\end{array}\right]$$

6c $${\varvec{K}}=\left[\begin{array}{cc}\boldsymbol{\Lambda }+{k}_{1}{\boldsymbol{\Phi }}^{T}\boldsymbol{\Phi }& -{k}_{1}{\boldsymbol{\Phi }}^{T}\\ {k}_{1}\boldsymbol{\Phi }& {k}_{1}\end{array}\right]$$

6d $${\varvec{F}}=\left({m}_{1}+{m}_{2}\right)g\left[\begin{array}{c}{\boldsymbol{\Phi }}^{T}\\ 0\end{array}\right]+\left[\left({c}_{1}v\dot{r}\left(vt\right)+{k}_{1}r\right)\left[\begin{array}{c}{\boldsymbol{\Phi }}^{T}\\ -1\end{array}\right]\right]$$

where ${\xi }_{i}$ is the $i$th modal damping ratio, ${\omega }_{i}$ is the $i$th undamped natural frequency, and $$\boldsymbol{\Phi }\left(vt\right)=\left[\begin{array}{cccc}{\varphi }_{1}\left(vt\right)& {\varphi }_{2}\left(vt\right)& \cdots & {\varphi }_{N}\left(vt\right)\end{array}\right]$$, $\boldsymbol{\Gamma }=\mathrm{diag}\left(2{\xi }_{1}{\omega }_{1},\cdot \cdot \cdot ,2{\xi }_{N}{\omega }_{N}\right)$, $\boldsymbol{\Lambda }=\mathrm{diag}\left({\omega }_{1}^{2},\cdot \cdot \cdot ,{\omega }_{N}^{2}\right)$. The coefficient matrices ${\varvec{M}}$, ${\varvec{C}}$, ${\varvec{K}}$ change continuously with time when the vehicle moves on the bridge.

The information of structural damage is covered in the dynamic response of the bridge. The gap between the predicted and the measured responses of the structure is minimized, by updating the damage parameters of the bridge model.

Vehicle parameters and road surface roughness are important to predict the response of VBI system, but the vehicle and road roughness information are usually unknown in practical engineering. The unknown variables in the VBI system model make it very difficult to predict vibration response of the bridge accurately, the model updating algorithm will be inaccurate for the above reason.

**Figure 2:**
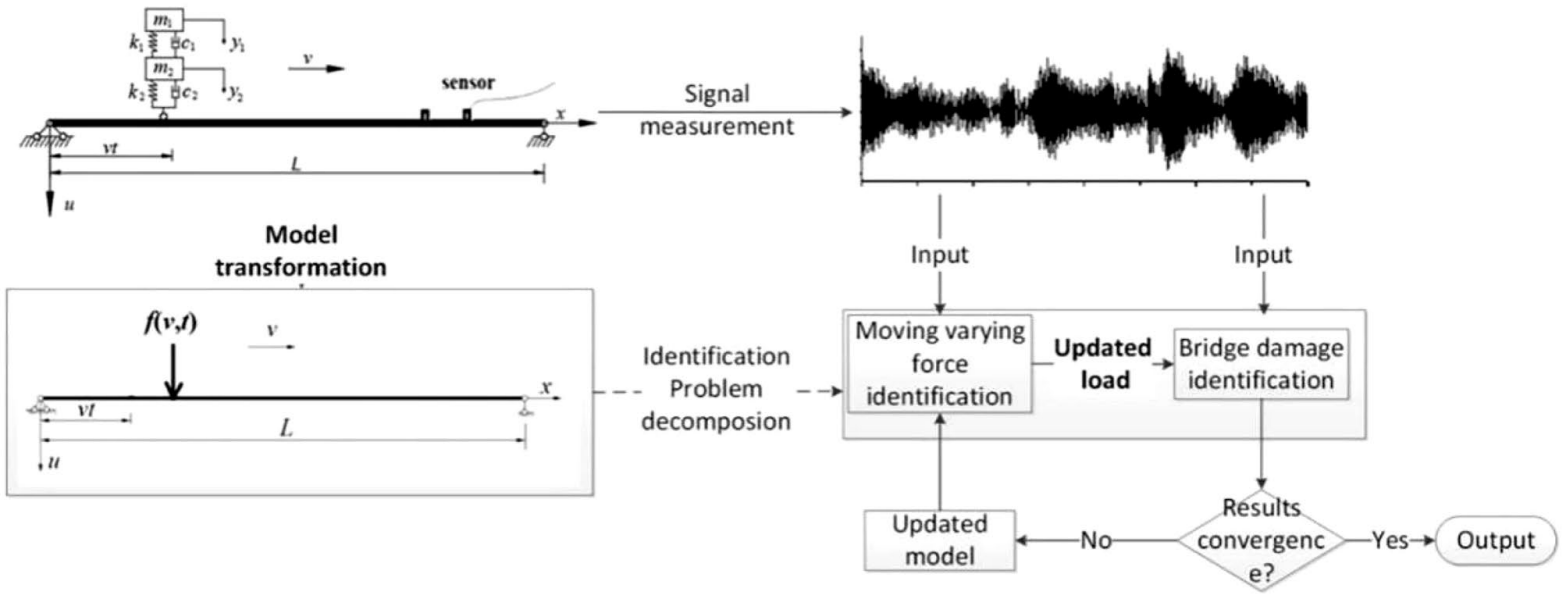
Framework for the conversion of damage identification.

Recognizing vehicle parameters and road roughness might be a solution of the problem, but this aim is also very hard to achieve. Therefore, in this paper, converting the classic VBI model to be a model that does not contain information of unknown vehicle parameters and road roughness is the basic idea. The VBI model is transformed into a bridge subject to a moving force, as shown in Fig. 2. The difficulties of measuring vehicle parameters and road roughness will be avoided if time-varying contact forces is identified.

The damage identification problem was decomposed into two sub problems: moving contact force identification and bridge damage identification under known loads. The vibration signals of the bridge are taken as the input of the moving contact force identification and bridge damage identification in turn. The results of the force identification provide more accurate loads to bridge damage identification, and the updating of the bridge model make the force identification more accurate. The iterative of moving contact force identification and damage identification will be redone until the change of the damage identification results are less than the limit.

### Moving-vehicle load identification based on extended Kalman filter

When the classic VBI model is transformed into a bridge vibration model under a moving contact force, a recursive algorithm based on EKF is proposed to identify the time-varying contact force $f\left(vt,t\right)$.

Based on non-destructive or known damage status of the bridge (not necessarily accurate), substituting Eq. (3) into Eq. (2) and applying the orthogonality conditions of mode shapes, then the dynamic equation of bridge is transformed into modal coordinates as

7 $$\begin{array}{cc}{\ddot{p}}_{i}\left(t\right)+2{\xi }_{i}{\omega }_{i}{\dot{p}}_{i}\left(t\right)+{\omega }_{i}^{2}{p}_{i}\left(t\right)={\varphi }_{i}\left(vt\right)f\left(vt,t\right),& i=\mathrm{1,2},...N\end{array}.$$

In order to reduce the dimension of the state vector, the modal truncation method is adopted, only the first three modal components, because the vibration response of the bridge structure mainly contains low-order modal components. The forced vibration equation is written into a matrix form

8 $$\ddot{{\varvec{p}}}\left(t\right)+\boldsymbol{\Gamma }\dot{{\varvec{p}}}\left(t\right)+\boldsymbol{\Lambda }{\varvec{p}}\left(t\right)={\boldsymbol{\Phi }}^{T}\left(vt\right)f\left(vt,t\right)$$

where ${\varvec{p}}\left(t\right)={\left[{p}_{1}\left(t\right),\cdot \cdot \cdot ,{p}_{N}\left(t\right)\right]}^{T}$, ${p}_{i}\left(t\right)$ is the $i$ th general modal coordinate of bridge structure.

The contact force $f$ to be solved can be assumed to be constant from the time $k$ to $k+1$, so the derivative of the contact force with time is assumed to be 0 between the time $k$ and $k+1$.

9 $$\dot{f}=0$$

However, $f$ is not a constant actually, so the contact force $f$ at time $k+1$ and $k$ are assumed to be related as followed:

10 $$f_{k + 1} = f_{k} + \eta_{k}$$

where a random variable ${\eta }_{k}$ is introduced in Eq. (10) to reflect the changes in force. ${\eta }_{k}$ is a zero mean random number.

In order to identify contact forces, the unknown force $f$ is covered in the augmented state vector of the EKF algorithm $\uptheta \left(\mathrm{t}\right)$:

11 $${\varvec{\theta}}\left(t\right)={\left[{\varvec{p}}\left(t\right) \dot{{\varvec{p}}}\left(t\right) f\left(vt,t\right)\right]}^{T}$$

The dynamics Eq. (8) can be represented as the corresponding state space formulation

12 $$\dot{{\varvec{\theta}}}\left(t\right)={\left[\begin{array}{ccc}\dot{{\varvec{p}}}\left(t\right)& {\boldsymbol{\Phi }}^{T}\left(vt\right)f\left(vt,t\right)-\boldsymbol{\Gamma }\dot{{\varvec{p}}}\left(t\right)-\boldsymbol{\Lambda }{\varvec{p}}\left(t\right)& 0\end{array}\right]}^{T}={{\varvec{g}}}_{f}\left({\varvec{\theta}},t\right)$$

Based on Eqs. (4) and (12) the corresponding state and observation equations of the EKF is written as:

13 $$\dot{{\varvec{\theta}}}\left(t\right)={{\varvec{g}}}_{f}\left({\varvec{\theta}},t\right)+{{\varvec{W}}}_{f}$$

14 $${{\varvec{y}}}_{f}\left({{\varvec{x}}}^{\mathbf{*}},t\right)={{\varvec{h}}}_{f}\left({\varvec{\theta}},{{\varvec{x}}}^{\mathbf{*}},t\right)+{{\varvec{V}}}_{f}$$

where ${{\varvec{y}}}_{f}\left({{\varvec{x}}}^{\boldsymbol{*}},t\right)$ is the response measurement (displacement, velocity, acceleration) at the measurement points ${{\varvec{x}}}^{\boldsymbol{*}}$, ${{\varvec{W}}}_{f}$ is the system noise, and is assumed to be zero mean multivariate Gaussian noises with covariance ${{\varvec{Q}}}_{f}$, the corresponding observation noise ${{\varvec{V}}}_{f}$ is zero mean multivariate Gaussian noises with covariance ${ {\varvec{R}}}_{f}$. The estimated value of the structural vibration response function ${{\varvec{h}}}_{f}\left({\varvec{\theta}},{{\varvec{x}}}^{\boldsymbol{*}},t\right)$ with different signal types are expressed as followed:

15 $${{\varvec{h}}}_{f}\left({\varvec{\theta}},{{\varvec{x}}}^{*},t\right)=\left\{\begin{array}{c}\sum_{i=1}^{N}{p}_{i}\left(t\right){\varphi }_{i}\left({{\varvec{x}}}^{*}\right),\quad if\, displacement\, is\, measured\\ \sum_{i=1}^{N}{\dot{p}}_{i}\left(t\right){\varphi }_{i}\left({{\varvec{x}}}^{*}\right),\quad if\, velocity\, is\, measured \\ \sum_{i=1}^{N}\left[{\varphi }_{i}\left(vt\right)f-2{\xi }_{i}{\omega }_{i}\dot{{p}_{i}}\left(t\right)-{\omega }_{i}^{2}{p}_{i}\left(t\right)\right]{\varphi }_{i}\left({{\varvec{x}}}^{*}\right),\quad if\, acceleration\, is\, measured\end{array}\right.$$

The EKF algorithm linearize the system equations around the previous state estimation with multivariate Taylor series expansion, the discretized recursive equations are obtained as followed:

16 $$\widetilde{{\varvec{\theta}}}\left(k+1\right)={{\varvec{A}}}_{f}\widehat{{\varvec{\theta}}}\left(k\right)$$

17 $$\widetilde{{\varvec{P}}}\left(k+1\right)={{\varvec{A}}}_{f}\widehat{{\varvec{P}}}\left(k\right){{\varvec{A}}}_{f}^{T}+{{\varvec{Q}}}_{f}\left(k+1\right)$$

18 $${{\varvec{K}}}_{f}\left(k+1\right)=\widetilde{{\varvec{P}}}\left(k+1\right){{\varvec{H}}}_{f}^{T}\left(k+1\right){\left[{{\varvec{H}}}_{f}\left(k+1\right)\widetilde{{\varvec{P}}}\left(k+1\right){{\varvec{H}}}_{f}^{T}\left(k+1\right)+{{\varvec{R}}}_{f}\left(k+1\right)\right]}^{-1}$$

19 $$\widehat{{\varvec{\theta}}}\left(k+1\right)=\widetilde{{\varvec{\theta}}}\left(k+1\right)+{{\varvec{K}}}_{f}\left(k+1\right)\left({{\varvec{y}}}_{f}\left(k+1\right)-{{\varvec{H}}}_{f}\left(k+1\right)\widetilde{{\varvec{\theta}}}\left(k+1\right)\right)$$

20 $$\widehat{{\varvec{P}}}\left(k+1\right)=\left({\varvec{I}}-{{\varvec{K}}}_{f}\left(k+1\right){{\varvec{H}}}_{f}\left(k+1\right)\right)\widetilde{{\varvec{P}}}\left(k+1\right)$$

where $\widehat{{\varvec{\theta}}}\left(k\right)$ is the estimated state vector of ${\varvec{\theta}}$ at time $k$, $\widetilde{{\varvec{\theta}}}\left(k+1\right)$ is the one-step predicted value at time $k+1$, $\widehat{{\varvec{P}}}\left(k\right)$ is the estimation error covariance at time $k$,$\widetilde{{\varvec{P}}}\left(k+1\right)$ is the error covariance matrix caused by the process of $\widetilde{\uptheta }\left(\mathrm{k}+1\right)$ relapcing real state $\uptheta \left(\mathrm{k}\right)$,$\mathrm{\Delta t}$ is the time step, ${\mathrm{y}}_{\mathrm{f}}\left(\mathrm{k}+1\right)$ is the response measurement (displacement, velocity, acceleration) at time $\mathrm{k}+1$, ${\mathrm{K}}_{\mathrm{f}}\left(\mathrm{k}+1\right)$ is the filter gain matrix at time $\mathrm{k}+1$, and the state transition matrix ${\mathrm{A}}_{\mathrm{f}}$ and observation matrix ${\mathrm{H}}_{\mathrm{f}}$ are obtained. ${\mathrm{Q}}_{\mathrm{f}}$ is the zero mean multivariate Gaussian noises covariance of the system noise ${\mathrm{W}}_{\mathrm{f}}$,${\mathrm{R}}_{\mathrm{f}}$ is the zero mean multivariate Gaussian noises covariance of the corresponding observation noise ${\mathrm{V}}_{\mathrm{f}}$, the state transition matrix ${\mathrm{A}}_{\mathrm{f}}$ is

21 $${{\varvec{A}}}_{f}\approx {\varvec{I}}+{{\varvec{A}}}_{g}\Delta t={\varvec{I}}+\frac{\partial {{\varvec{g}}}_{f}\left({\varvec{\theta}},t\right)}{\partial{\varvec{\theta}}}\Delta t$$

where the expression ${{\varvec{A}}}_{g}$ is shown in Appendix 1. The estimated value of the time-varying moving load will be obtained from the recursive process of (16) to (20) with the bridge vibration signals, assuming that the state of the bridge is known already.

### Bridge damage identification based on extended Kalman filter

The extended Kalman filter is an efficient recursive algorithm used to estimate the optimal state of a linear system. As a recursive optimization algorithm, EKF is also useful to the bridge damage identification. The structural damage parameter $\boldsymbol{\alpha }$ is covered in the augmented state vector of the EKF algorithm ${\varvec{\theta}}(t)$:

22 $${\varvec{\theta}}(t)={\left[{\varvec{p}}\left(t\right) \dot{{\varvec{p}}}\left(t\right) \begin{array}{c}\boldsymbol{\alpha }\end{array}\right]}^{T}$$

where $\boldsymbol{\alpha }=\left[{\alpha }_{1},\dots ,{\alpha }_{{n}_{e}}\right]$ is the damage parameters of the bridge elements, ${n}_{e}$ is the number of elements.

Refer to common description methods of bridge damage^14,32^, the reduction of the flexural stiffness ${\alpha }_{i}$ is introduced as the beam damage parameter

23 $$\alpha_{i} = \left( {E_{i}^{0} I_{i}^{0} - E_{i}^{d} I_{i}^{d} } \right)/E_{i}^{0} I_{i}^{0}$$

where ${E}_{i}^{0}$ and ${E}_{i}^{d}$ are elastic modulus of the undamaged and damaged beam of ith element, respectively. ${I}_{i}^{0}$ and ${I}_{i}^{d}$ are inertial moment of the undamaged and damaged beam of ith element, respectively.${E}_{i}^{0}{I}_{i}^{0}$,${E}_{i}^{d}{I}_{i}^{d}$ are flexural stiffness of the undamaged and damaged beam of ith element, respectively. ”

The damage parameter ${\alpha }_{i}$ is a constant, so the continuous system equation of the EKF is expressed as:

24 $$\dot{{\varvec{\theta}}}\left(t\right)={\left[\begin{array}{ccc}\dot{{\varvec{p}}}\left(t\right)& {\boldsymbol{\Phi }}^{T}\left(vt\right)f\left(vt,t\right)-\boldsymbol{\Gamma }\dot{{\varvec{p}}}\left(t\right)-\boldsymbol{\Lambda }{\varvec{p}}\left(t\right)& {0}_{1\times {n}_{e}}\end{array}\right]}^{T}={{\varvec{g}}}_{\alpha }({\varvec{\theta}},t)$$

The system state and measurement equation based on EKF algorithm is represented as

25 $$\dot{{\varvec{\theta}}}\left(t\right)={{\varvec{g}}}_{\alpha }\left({\varvec{\theta}},t\right)+{{\varvec{W}}}_{\alpha }$$

26 $${\varvec{y}}_{\alpha } \left( {{\varvec{x}}^{*} ,t} \right) = {\varvec{h}}_{\alpha } \left( {{\varvec{\theta}},{\varvec{x}}^{*} ,t} \right) + {\varvec{V}}_{\alpha }$$

where ${{\varvec{y}}}_{\alpha }\left({{\varvec{x}}}^{\boldsymbol{*}},t\right)$ is the response measurement at the measurement point ${{\varvec{x}}}^{\boldsymbol{*}}$, ${{\varvec{W}}}_{\alpha }$ and ${{\varvec{V}}}_{\alpha }$ are the process noise and the observe noise, which are assumed to be zero mean multivariate Gaussian noises with covariance ${{\varvec{Q}}}_{\alpha }$ and ${{\varvec{R}}}_{\alpha }$, respectively. The estimated value of the structural vibration response function ${{\varvec{h}}}_{f}\left({\varvec{\theta}},{{\varvec{x}}}^{\boldsymbol{*}},t\right)$ with different signal types are expressed as followed:

27 $${{\varvec{h}}}_{\alpha }\left({\varvec{\theta}},{{\varvec{x}}}^{*},t\right)=\left\{\begin{array}{c}\sum_{i=1}^{N}{p}_{i}\left(t\right){\varphi }_{i}\left({{\varvec{x}}}^{*}\right),\quad if\, displacement\, is\, measured\\ \sum_{i=1}^{N}{\dot{p}}_{i}\left(t\right){\varphi }_{i}\left({{\varvec{x}}}^{*}\right),\quad if\, velocity\, is\, measured \\ \sum_{i=1}^{N}\left[{\varphi }_{i}\left(vt\right)f-2{\xi }_{i}{\omega }_{i}\dot{{p}_{i}}\left(t\right)-{\omega }_{i}^{2}{p}_{i}\left(t\right)\right]{\varphi }_{i}\left({{\varvec{x}}}^{*}\right),\quad if\, acceleration\, is\, measured\end{array}\right.$$

The state variables ${\varvec{p}}$ and $\dot{{\varvec{p}}}$ coupled nonlinearly with the structural parameter $\boldsymbol{\alpha }$, for this reason, the estimation problem is nonlinear. The Taylor series expansion to is used to linearize the state equations and observation equations of the system. According to the EKF algorithm, the discretization equations for discretization and linearization of the ${t}_{k}$ time system can be obtained.

28 $$\widetilde{{\varvec{\theta}}}\left(k+1\right)=\widehat{{\varvec{\theta}}}\left(k\right)+\left({\int }_{t}^{t+\Delta t}{{\varvec{g}}}_{\alpha }\left({\varvec{\theta}},t\right)\mathrm{d}t\right)\Delta t$$

29 $$\widetilde{{\varvec{P}}}\left(k+1\right)={{\varvec{A}}}_{\alpha }\widehat{{\varvec{P}}}\left(k\right){{\varvec{A}}}_{\alpha }^{T}+{{\varvec{Q}}}_{\alpha }\left(k+1\right)$$

30 $${{\varvec{K}}}_{\alpha }\left(k+1\right)=\widetilde{{\varvec{P}}}\left(k+1\right){{\varvec{H}}}_{\alpha }^{T}\left(k+1\right){\left[{{\varvec{H}}}_{\alpha }\left(k+1\right)\widetilde{{\varvec{P}}}\left(k+1\right){{\varvec{H}}}_{\alpha }^{T}\left(k+1\right)+ {{\varvec{R}}}_{\alpha }\left(k+1\right)\right]}^{-1}$$

31 $$\widehat{{\varvec{\theta}}}\left(k+1\right)=\widetilde{{\varvec{\theta}}}\left(k+1\right)+{{\varvec{K}}}_{\alpha }\left(k+1\right)\left({{\varvec{y}}}_{\alpha }\left(k+1\right)-{{\varvec{h}}}_{\alpha }\left[\widetilde{{\varvec{\theta}}}\left(k+1\right)\right]\right)$$

32 $$\widehat{{\varvec{P}}}\left(k+1\right)=\left({\varvec{I}}-{{\varvec{K}}}_{\alpha }\left(k+1\right){{\varvec{H}}}_{\alpha }\left(k+1\right)\right)\widetilde{{\varvec{P}}}\left(k+1\right)$$

where parameters are the same as Eqs. (16)–(20). The state transition matrix ${{\varvec{A}}}_{\alpha }$ is

33 $${{\varvec{A}}}_{\alpha }\approx {\varvec{I}}+{{\varvec{A}}}_{B}\Delta t={\varvec{I}}+\frac{\partial {{\varvec{g}}}_{\alpha }\left({\varvec{\theta}},t\right)}{\partial{\varvec{\theta}}}\Delta t$$

where the expression ${{\varvec{A}}}_{B}$ of is shown in Appendix 3.

The initial estimates of the state vector and the error covariance matrix, ${\widehat{{\varvec{\theta}}}}_{0}$ and ${\widehat{{\varvec{P}}}}_{0}$, need to be given before the EKF recursion. The state vector includes two parts, the state of motion and the state of damage. The initial state of the motion is static and the initial value of the damage parameter is set as 0. It is consistent with the practical engineering. The numerical examples show that the initial value of the state vector has less effect on convergence of a stable EKF process. It is possible to identify the structural damage condition even without an accurate initial state, which is a great advantage in applying the EKF method to the field of structural damage identification.

In the current recursive step, the modal parameters (natural frequency, mode shape) of the structure is obtained by the damage parameters of the sensitivity matrix. The predicted value of the structural response is obtained through the modal superposition method. The state vector is updated by reducing the difference between the predicted and the measured response with the EKF algorithm, the best estimate of the structural damage condition will be got as the final result.

The classical EKF algorithm provides an optimal or suboptimal estimate ${\widehat{{\varvec{\theta}}}}_{k}$ with the least mean square error, which is the solution to the unconstrained $l2$-norm minimization problem.

34 $$\underset{\widehat{{\varvec{\theta}}}(k)}{\mathrm{min}}{\mathrm{E}}_{{\varvec{\theta}}(k)|{\varvec{z}}(k)}\left[{\Vert {\varvec{\theta}}(k)-\widehat{{\varvec{\theta}}}(k)\Vert }_{2}^{2}\right]$$

The structure suffered local damages, therefore, only a few damage parameters at the local damage elements are non-zeros, which means the distribution of damage parameters is sparse. The prior sparse feature of damage parameters is usually introduced in the form of $l1$-norm regularization^33^ to improve the ill-posedness of the inverse problem. By the pseudo-measurement technique, the author^34^ proposed a damage identification algorithm based on EKF combined with $l1$-norm regularization, which is also used in this paper.

This PM can be rewritten as

35 $$0 = \overline{\user2{H}} \cdot {\varvec{\theta}}_{K} - \varepsilon { }$$

where $\overline{{\varvec{H}} }=\left[{0}_{1\times N},{0}_{1\times N},sign\left({\alpha }_{1}\right),\cdot \cdot \cdot ,sign\left({\alpha }_{m}\right),{0}_{1\times N}\right]$, $sign\left({\alpha }_{i}\right)$ denotes the sign function of damage parameter ${\alpha }_{i}$,

36 $$sign\left( {\alpha_{i} } \right) = \left\{ {\begin{array}{*{20}c} { 1,\quad if \alpha_{i \ge 0} } \\ { - 1,\quad if \alpha_{i < 0} } \\ \end{array} } \right.$$

In pseudo measurement process, $\varepsilon$ serves as the measurement noise, the role of the covariance ${{\varvec{R}}}_{\varepsilon }$ is similar with regularization parameter, and the optimal value of the covariance ${{\varvec{R}}}_{\varepsilon }$ can be determined by the L-curve method^35^ to balance the response residuals and $l1$-norm regularization constraints.

### Step-by-step recursive algorithm based on EKF with ${\varvec{l}}1$-norm

Considering the interference from measurement noises, the identification convergence conditions in this paper are set as:

37 $$\left|{\alpha }_{i}^{u}-{\alpha }_{i-1}^{u}\right|<0.005, \left|{\alpha }_{i}^{d}-{\alpha }_{i-1}^{d}\right|<0.01$$

where ${\alpha }_{i}^{d}$ and ${\alpha }_{i-1}^{d}$ are the identification results of damage parameters in damaged elements at last and second last calculation processes, respectively, and ${\alpha }_{i}^{u}$ and ${\alpha }_{i-1}^{u}$ are the identification results of damage parameters in undamaged elements at last and second last calculation processes, respectively.

Therefore, the steps of step-by-step identification recursive algorithm based on EKF are summarized in Table 1.

**Table 1:**
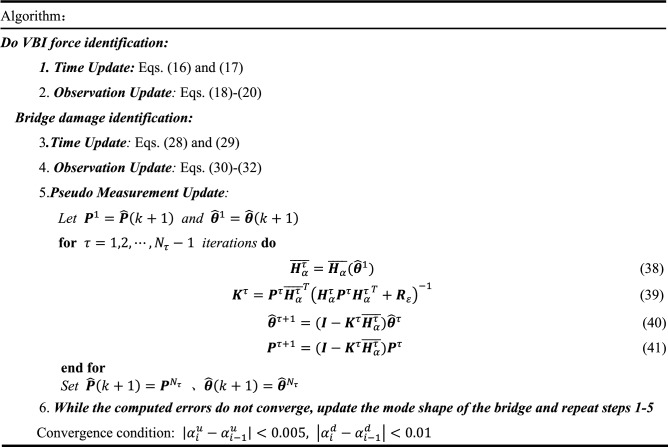
Step-by-step identification method based on EKF with l1-norm regularization.

In this paper, the EKF of linearization method is used to deal with nonlinear models. In order to improve the accuracy of the results, some nonlinear Kalman filters such as Embedded cubature Kalman filter method^36^ could be used in the future research. The adaptive estimation methods^37^ could be used for the system and measurement noise covariance matrices. In practical engineering application, the system noise and measurement noise might be non-Gaussian distributed caused by measurement outliers, some recent robust Kalman filters^38^ could be tried in the future jobs.

The PC configuration used for calculations is as following: OS of windows; Core(TM) of i7-8565U; CPU of 1.80 GHz; Memory of 8 GB. The time for the moving force identification of each iteration step is 6.06 s. The time for the bridge damage identification of each iteration step is 4.12 s.

## Numerical studies

### Numerical model

**Figure 3:**
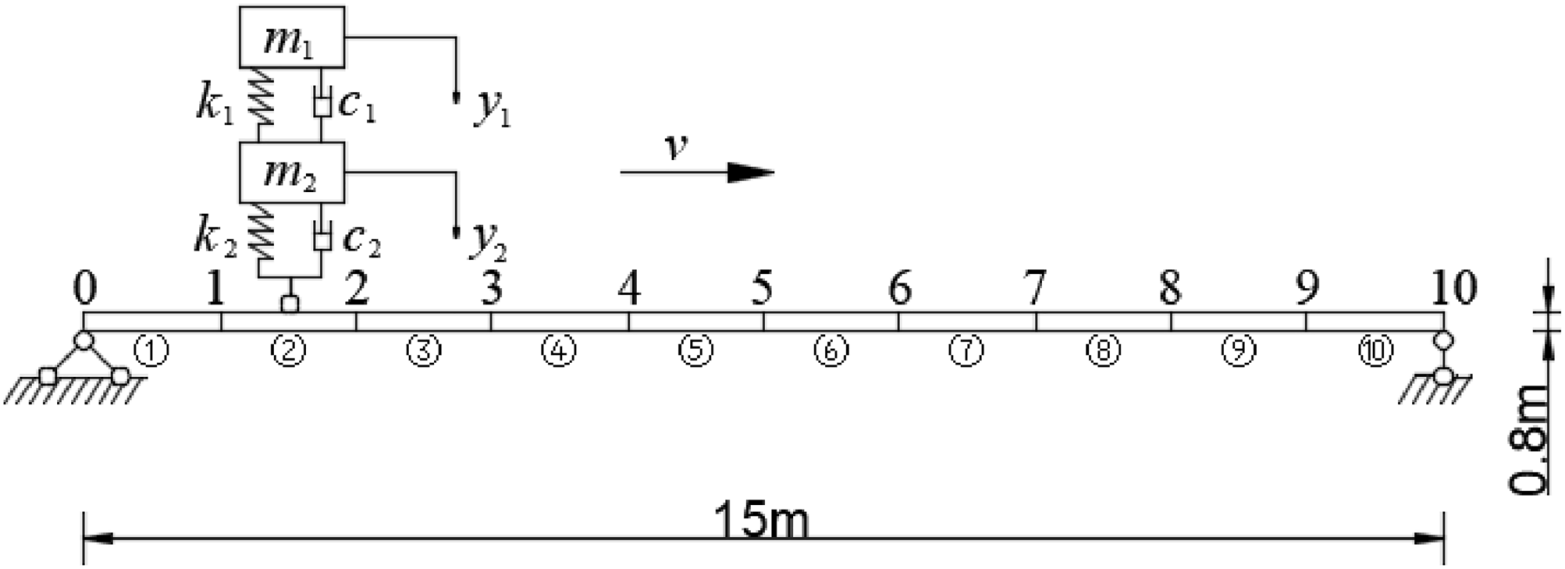
Simple supported beam and the distribution of measurement points.

In order to investigate the applicability of the proposed algorithm for bridge damage identification, a simply supported beam bridge is used as the numerical example^39^. The bridge model is shown in Fig. 3, the physical parameters of the beam are $L=15\mathrm{m}$, $I={0.512\mathrm{m}}^{4}$, $E=31\mathrm{GPa}$, and $\rho =2400\mathrm{kg}/{\mathrm{m}}^{3}$. The modal damping ratio of the first three orders is $\xi =0.02$. The parameters of the 1/4 vehicle model are as follows: body mass ${m}_{1}=2\times {10}^{4}\mathrm{kg}$, sum of suspension and tire mass ${m}_{2}=100\mathrm{kg}$, stiffness of the suspension spring is ${{k}_{1}=3\times 10}^{6}\mathrm{N}/\mathrm{m}$, the damping coefficient of the suspension ${{c}_{1}=3\times 10}^{6}N/\mathrm{m}$, the equivalent stiffness of the tire is ${{k}_{2}=2.85\times 10}^{6}\mathrm{N}\cdot \mathrm{s}/\mathrm{m}$, ${c}_{2}=0$.

The beam is divided into 10 Euler–Bernoulli beam elements evenly with 11 nodes and the sensors are installed on nodes 1 to 9 to measure the vertical displacement of nodes under moving load. Each beam element node in the vertical plane has 3 DOFs.

The vibration signal is sampled at a frequency of 1000 Hz. The measured signal ${\varvec{y}}\left({{\varvec{x}}}^{\boldsymbol{*}},t\right)$ is simulated by the original vibration signals ${\varvec{u}}\left({\varvec{\theta}},{{\varvec{x}}}^{\boldsymbol{*}},t\right)$ add with Gaussian white noise ${\varvec{V}}$.

42 $${\varvec{y}}\left({{\varvec{x}}}^{*},t\right)={\varvec{u}}\left({\varvec{\theta}},{{\varvec{x}}}^{*},t\right)+{\varvec{V}}$$

The level of measurement noise ${\varvec{V}}$ can be represented by the signal-to-noise ratio (SNR) of dB.

43 $$\mathrm{SNR}=10\times \mathrm{lg}\left(\frac{sp}{np}\right)$$

where $sp$ is the signal power, and $np$ is the noise power.

The vertical vibrations of the vehicle and the bridge are ignored before the vehicle enters the bridge, and the vehicle moving speed $v$ is set as a fixed value. Different factors such as the vehicle speed, the damage cases, the measurement noise, and the road roughness level are considered in order to make sure the damage positions and the damage extent. The single and multiple damage cases are considered, as shown in Table 2. In these two cases, three noise levels, 25, 30 and 35 dB and three vehicle speed 10 m/s, 15 m/s and 20 m/s are analyzed.

**Table 2:** Two local damage cases.

	Case 1	Case 2		
Damage element	6	3	6	9
Damage parameter	0.3	0.2	0.3	0.15

### Damage identification in different damage cases

#### Identification of moving contact force and bridge damage on smooth road surface

In order to demonstrate the affection of the iterative process to the identification results, the case of smooth road surface is analyzed.

The measurement noise level is 30 dB, and the vehicle passes the bridge with the speed $v=15\mathrm{ m}/\mathrm{s}$.

**Figure 4:**
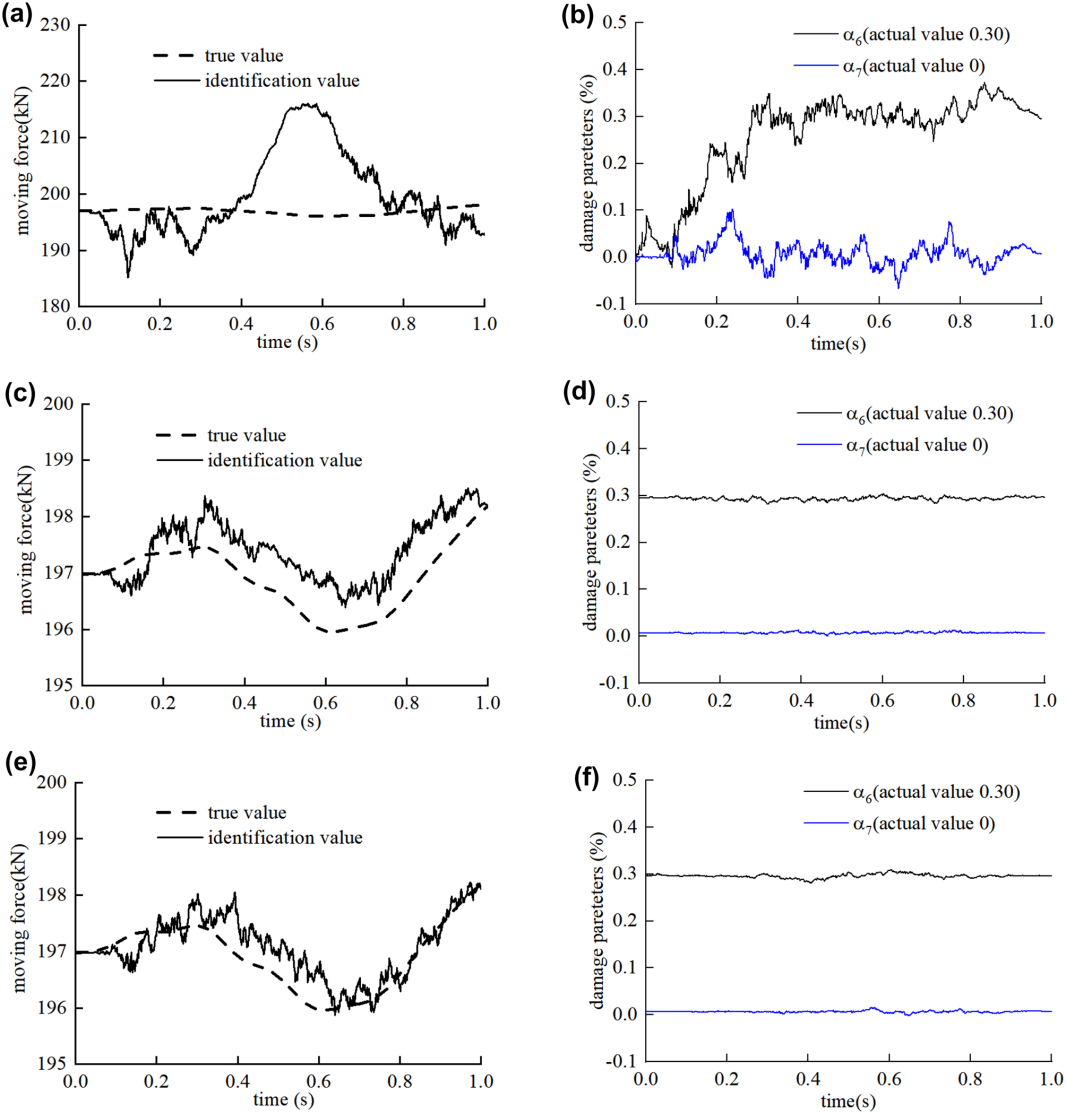
The identification results of the contact moving force in the (**a**) initial iterative process, (**c**) second iterative process, (**e**) third iterative process and the identification results of damage parameters at the node No. 6 and No. 7 in the (**b**) initial iterative process, (**d**) second iterative process, (**f**) third iterative process.

The identification results of three iterations in the single damage case are shown in Fig. 4. In the initial iteration process, the initial state of the bridge is assumed undamaged. The contact force identification fluctuates around the true value because of the inaccurate model. The maximum relative error of the identified moving contact force is close to 10% as shown in the Fig. 4a. Then the identified moving contact force is further used to identify the bridge damages as a known load. The convergence curves of damage parameter of the damaged element No. 6 and the undamaged element No. 7 are shown in Fig. 4b. The results of damage parameter converge to the true value quickly even if the moving contact force is not accurate.

In the second iteration process, the bridge model was updated with the identified damage parameters from the former step. The maximum error of the moving force identification is significantly reduced from 10 to 1% since the revised bridge model is closer to the real bridge model. In the third iteration process, the accuracy of the moving force identification results is slightly improved, and the identification results of bridge damage are basically equal to the real value with high accuracy.

**Figure 5:**
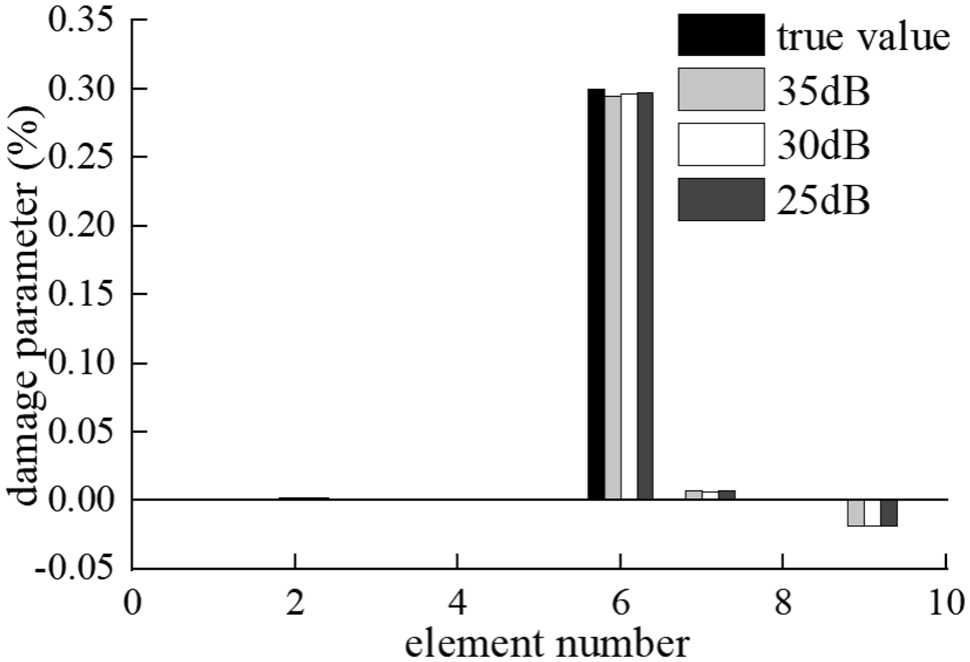
Result of damage identification in single damage case on the smooth road surface.

**Figure 6:**
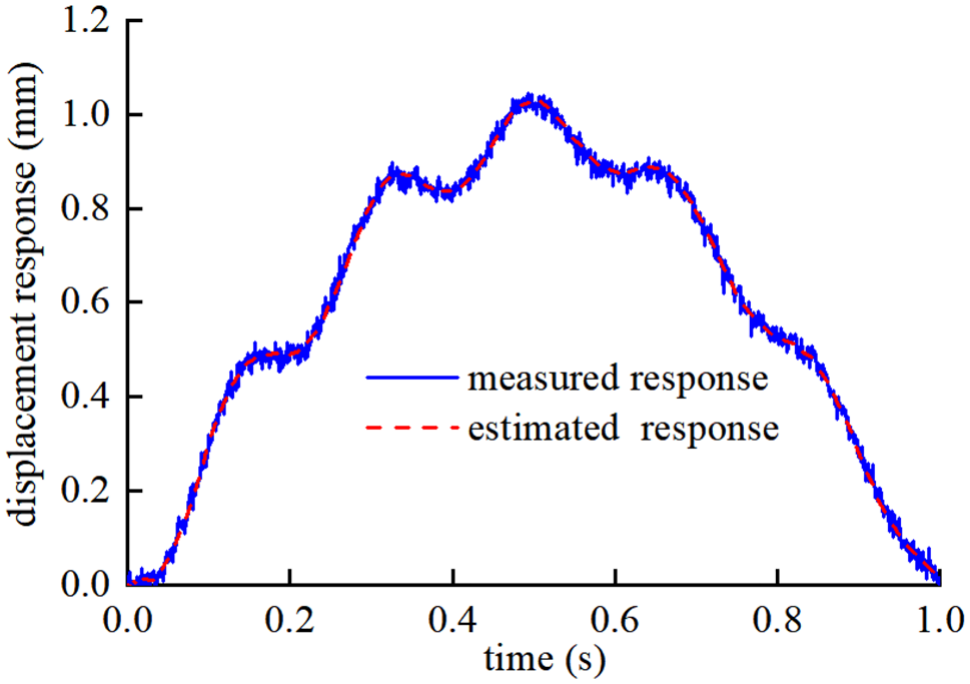
Displacement response tracking diagram of element No.6.

The results of the damage identification are of convergence after three iterations. As shown in Fig. 5, the results show a maximum discrepancy of 0.58% for the identification of the damaged elements stiffness and 1.89% for the identification of the undamaged elements stiffness. Figure 6 shows the identified structural response of the third iteration. The results show that the proposed algorithm suppresses noises effectively and tracks the true response signals accurately.

**Figure 7:**
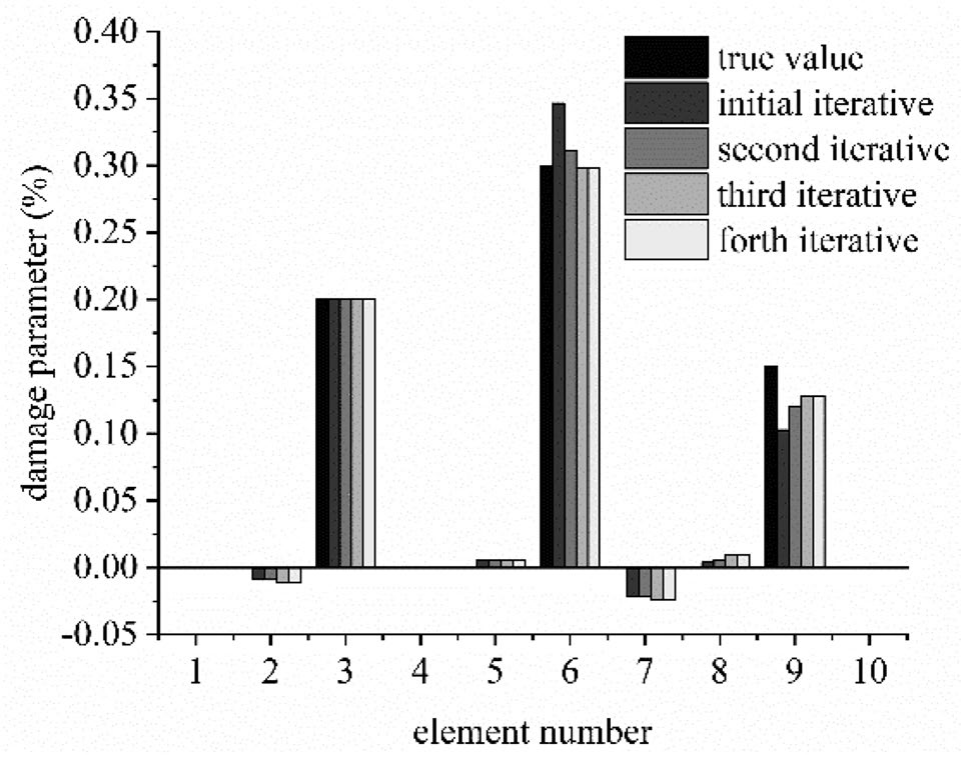
Damage identification result for each iterative process under noise level SNR = 30 dB.

The results of damage identification in the multiple damage case are shown in Fig. 7. The convergence speed of the damaged parameters in the multi-damage case is slower than that of the single damage case, but the convergence speed is still rapid. To the damaged elements, the maximum relative error of damaged element stiffness is already less than 0.59% at the third recursive step.

**Figure 8:**
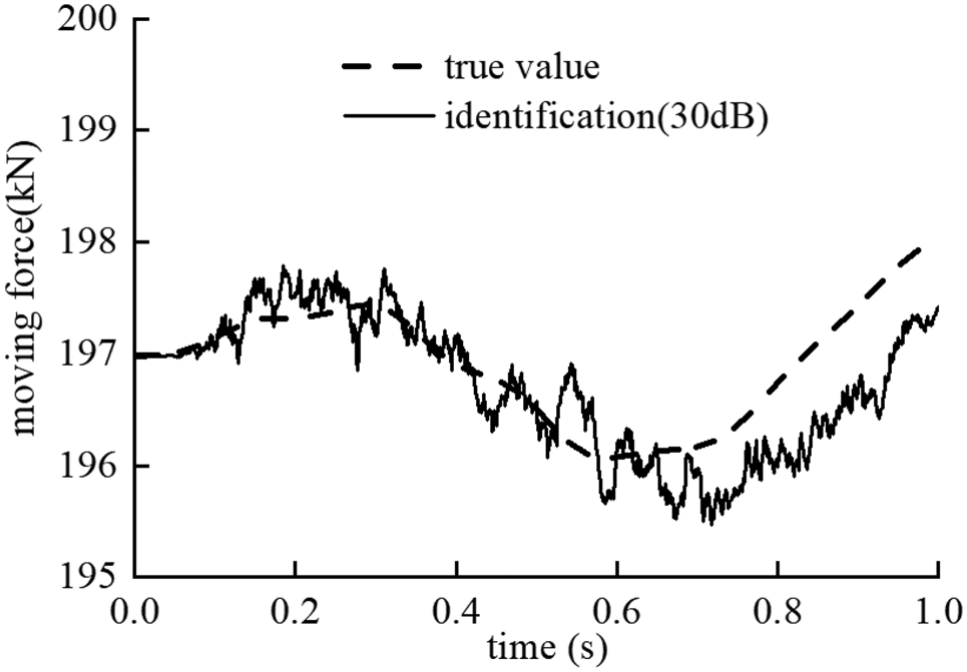
Identification result of moving force in the last iterative process.

The identification results of moving contact force in the last iterative process are shown in Fig. 8. Similar to the single damage case, the maximum relative error of the moving contact force between the estimated value and the real value is less than 1%. The method proposed in this paper is effective to identify the damage in the bridge both in the single-damage case and in the multi-damage case.

The $l1$-norm regularization is very important in the calculate progress. With the use of $l1$-norm regularization, the results of the undamaged element stiffness fluctuate more slightly, and converge to the true values quickly. On the other word, it is possible that the damage identification is not converge without $l1$-norm regularization, because the ill-posedness of damage inversion is stronger than load inversion.

It will take three or four iteration progresses for the multiple and single damage cases to achieve the accurate results. The convergence speed of the damage parameters in the multi-damage case is slightly slower than that of the single damage case. But for the multi-damage case, the maximum relative error of identified damaged element stiffness is already less than 0.59% at the third recursive step.

#### Identification of moving contact force and bridge damage considering the road roughness.

The same numerical example as for section “Identification of moving contact force and bridge damage on smooth road surface” is used to study the influence of road roughness. The bridge surface condition Class A, B and C are considered in accordance with the ISO specification Classes A-C, which can be benchmarked against good, average, and poor separately. In order to compare the identification results between simulated condition (like A, B, C) and the ideal condition, smooth road surface is considered as a reference standard.

**Figure 9:**
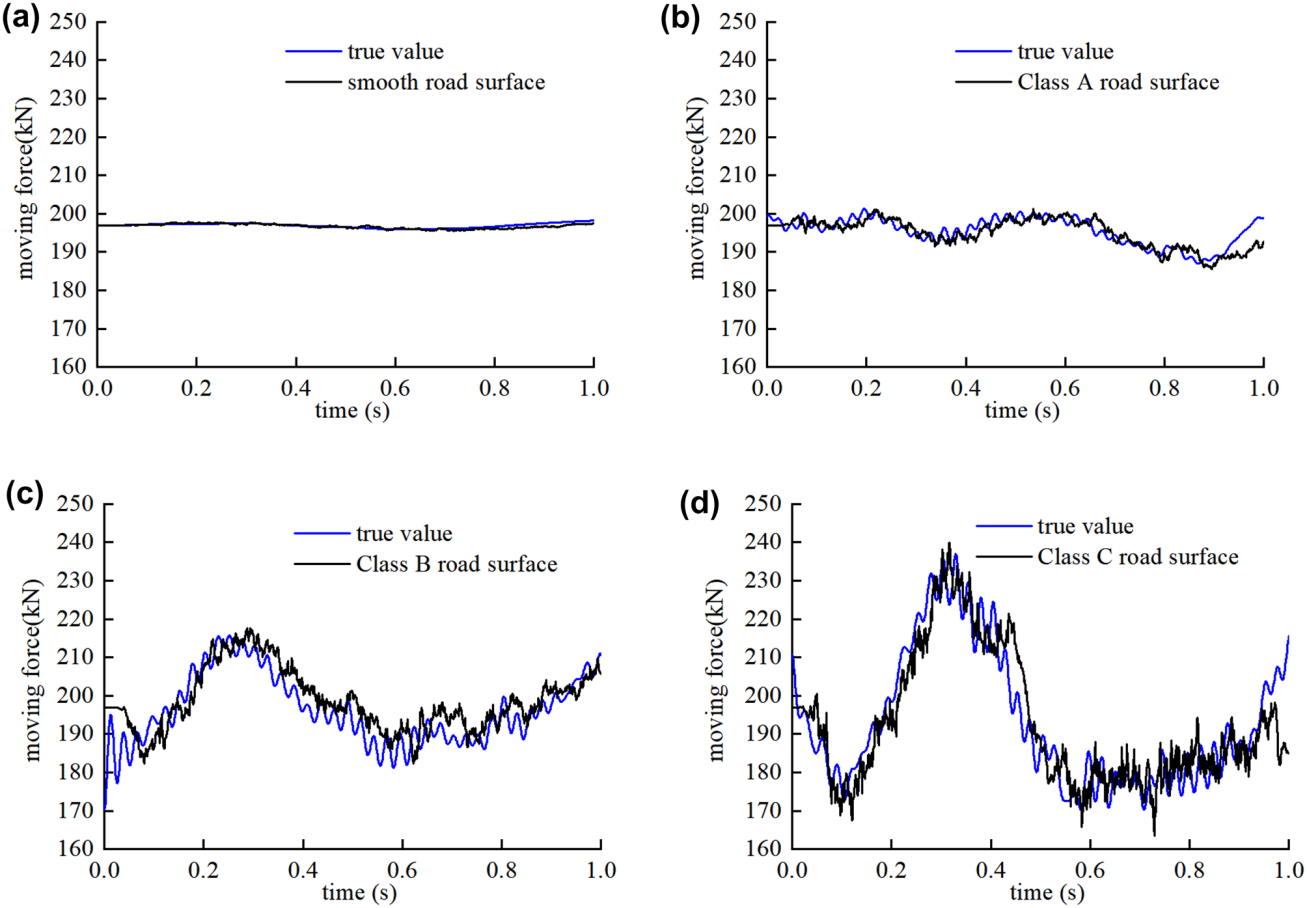
Identification results of moving force on different classes of road surface on (**a**) smooth road surface, (**b**) Class A road surface, (**c**) Class B road surface and (**d**) Class C road surface.

**Figure 10:**
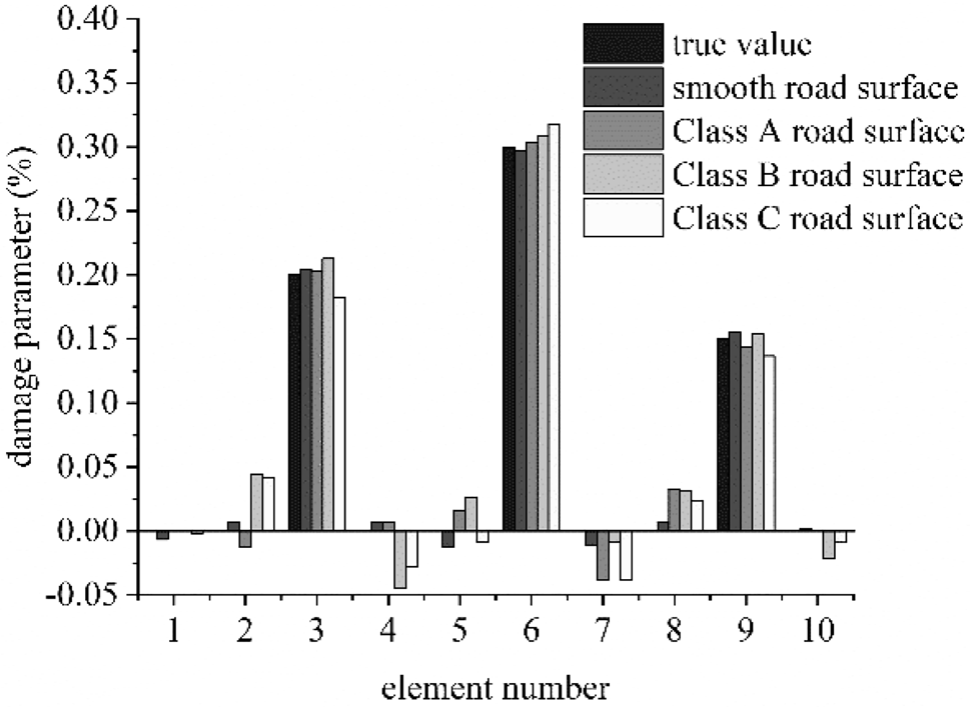
Damage identification results on different classes of road surface.

Figures 9 and 10 show the moving contact force and damage identification results in multi-damage case when the road surface exhibits different roughness levels, respectively.

The moving contact force is the only one exciting source on the smooth road, but the road roughness is another important exciting source of the VBI system when the road is rough. With the improvement of the road roughness level, the proposed method is more effective and the identification results of the moving force is more accurate. The road roughness is random and the contact force between the vehicle and the bridge will increase with the poorer of the road surface, as shown in the Fig. 9. But the moving contact force can still be identified accurately even for the Class-C roads surface with the proposed algorithm, the maximum relative error of the contact force identification is less than 2%. The maximum relative errors of the contact force identification for the smooth, Class-A and Class-B road surface are even smaller, which are 0.58%, 0.81%, 1.59% respectively.

Based on the accurate identification of the moving contact force, the damage positions and extent are identified accurately after several iterative progresses. The identification results show a maximum discrepancy of 0.58%, 0.81%, 1.59%, 2.59% for the damaged elements stiffness and 1.28%, 3.81%, 4.42%, 4.16% for the undamaged element stiffness when the class of the road surface is smooth, Class A, B and C, respectively.

As shown in this example, the information of the road roughness and vehicle are both unknown, but the problem are perfectly solved with the proposed algorithm, the identification results of the moving force and damage parameter are accurate with different road roughness levels.

The maximum relative errors of the damage identification results rise with the poorer of the road surface. But all the maximum relative errors are less than 5%, thus, the proposed algorithm is insensitive to the road roughness. The method is effective to identify damage and the moving contact force with different classes of roughness.

#### Identification of moving contact force and bridge damage with different noise level

The vibration response of the bridge used in this section is the displacement signal and the SNR is 35 dB, 30 dB, 25 dB, respectively. The influence of noise level is studied with the numerical example same as section “Identification of moving contact force and bridge damage on smooth road surface”. The vehicle passes the simply supported beam bridge with the speed of $v=15\mathrm{ m}/\mathrm{s}$. The roughness of road surface is set to be Class A, and the multi-damage case is considered.

**Figure 11:**
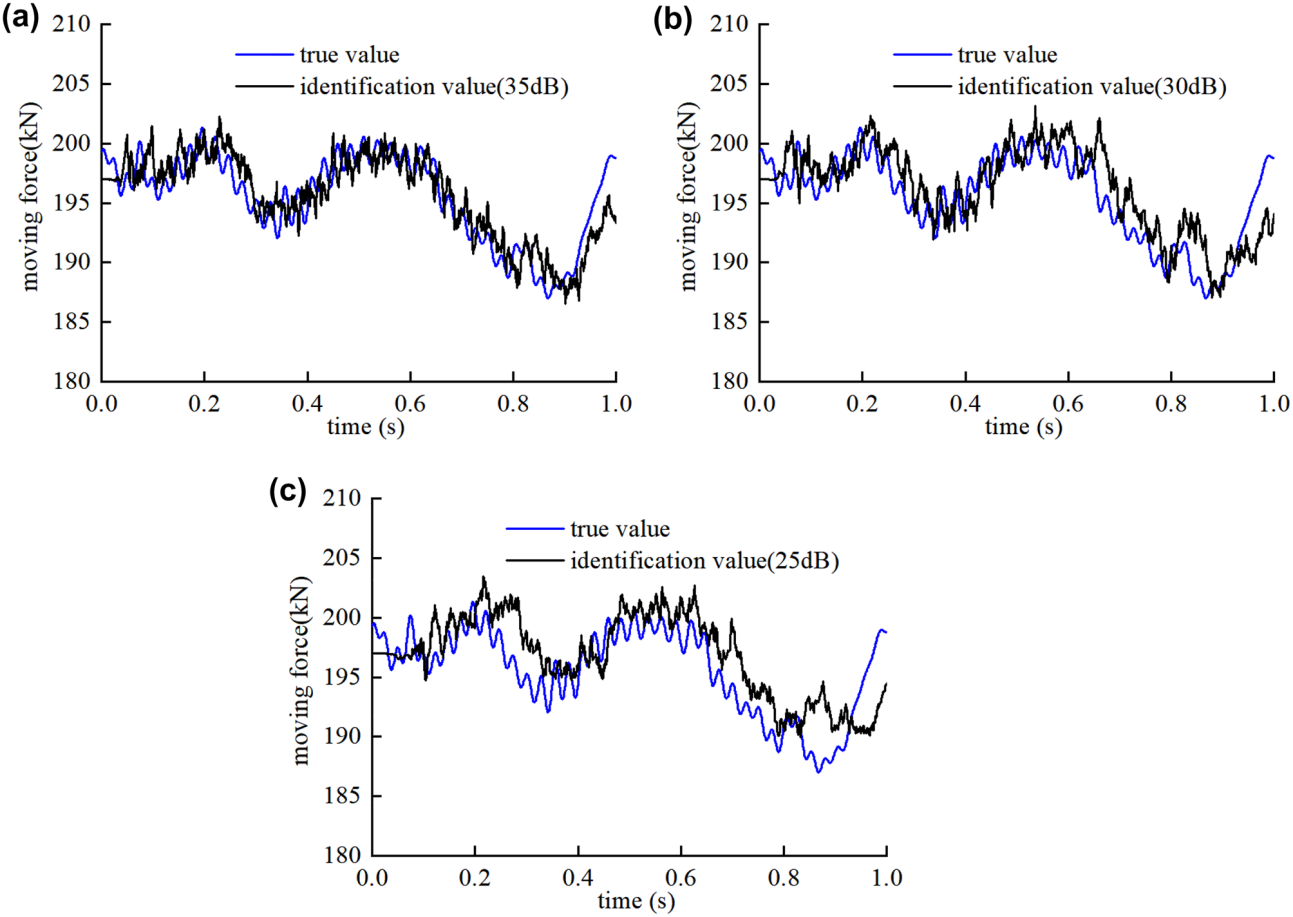
Identification results of moving force on Class-A road surface in multi-damage case under (**a**) 35 dB, (**b**) 30 dB and (**c**) 25 dB.

**Figure 12:**
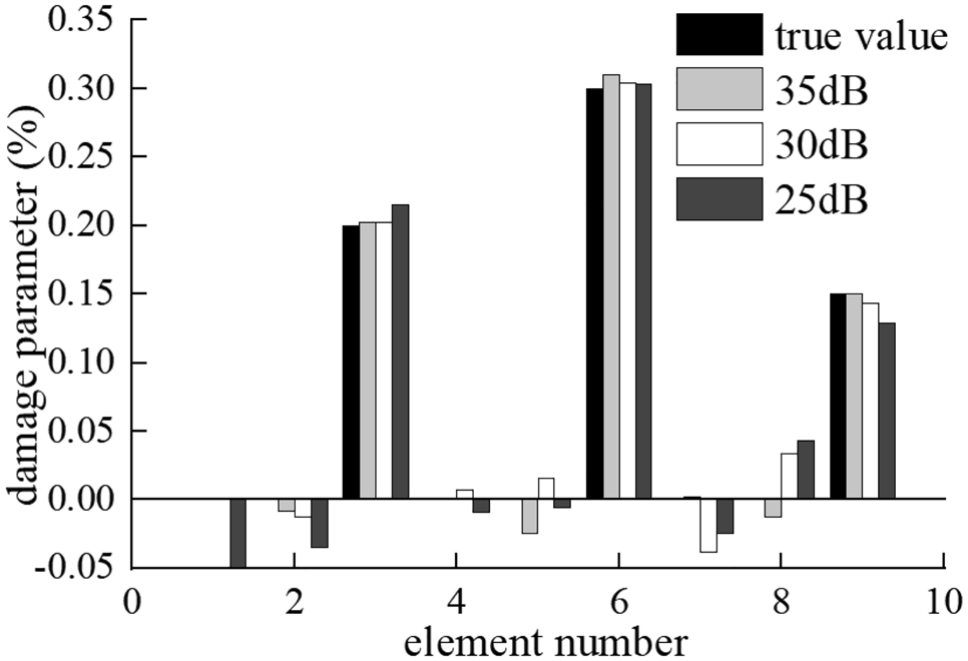
Damage identification result on Class A road surface with the speed of 15 m/s.

The identification results of moving force and the bridge damage in multi-damage case are shown in Figs. 11, 12. The damage position is accurately identified even when the SNR rises to 25 dB.

As shown in Fig. 11, the accuracy of the moving contact force identification reduces slightly, the average relative error is 0.79%, 0.99% and 1.16% for 35 dB, 30 dB and 25 dB, respectively. The accuracy of the moving contact force identification reduces with the increase of noise, but all the maximum relative errors are less than 3.45%.

As can be seen in the Fig. 12, the results show the maximum discrepancy of 1.88%, 2.53%, 4.13% for the identification of the damaged elements stiffness and 3.15%, 2.39%, 3.57% for the undamaged element stiffness when the noise level is 35 dB, 30 dB and 25 dB, respectively.

Based on the above data, the maximum relative errors of the moving contact force and the damage identification are both less than 5%, the results are accurate with different noise levels. The algorithm shows good robustness and convergence with different noise level, as both the moving contact force and the damage identification converge to their exact values rapidly even if the SNR reaches 25 dB. It is difficult to obtain accurate identification results without the $l1$-norm regularization by suppressing noise interference and introducing prior information.

#### Structure damage identification with different vehicle speeds

**Figure 13:**
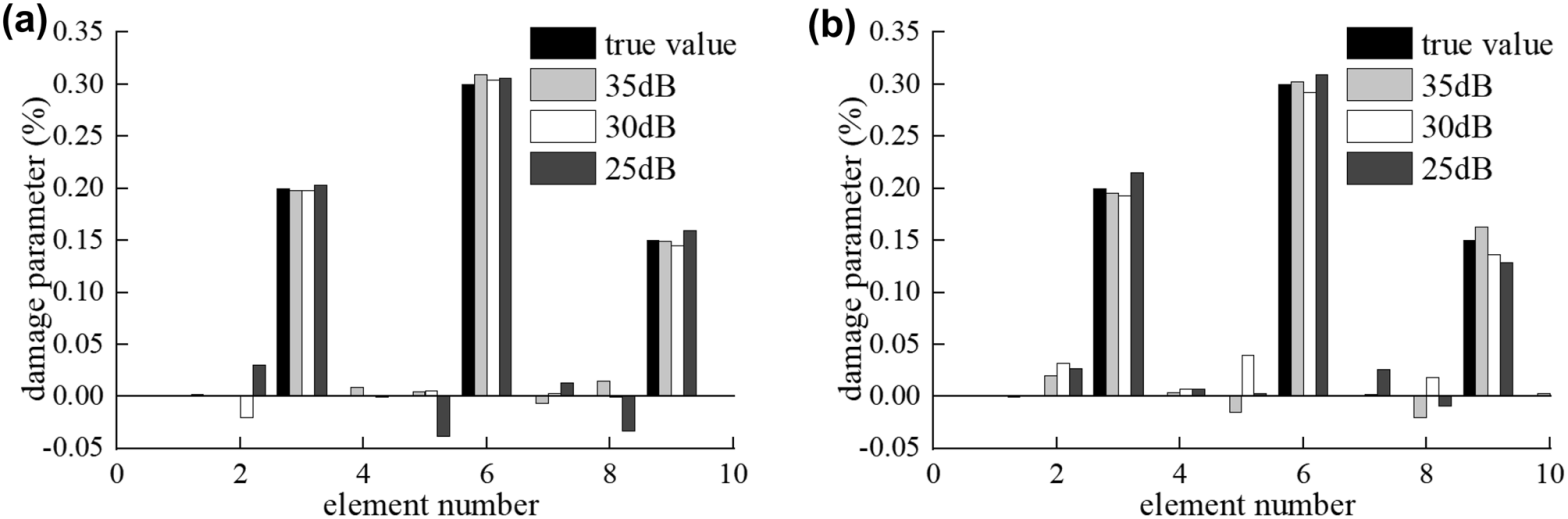
Identification results of moving force and structure damage on Class A road surface (**a**) with the speed of 10 m/s and (**b**) with the speed of 20 m/s.

The parameters of the bridge and the vehicle are the same as for section “Identification of moving contact force and bridge damage on smooth road surface”. The vehicle moved on the bridge with the speed of $v=20\mathrm{ m}/\mathrm{s}$, $v=15\mathrm{ m}/\mathrm{s}$, $v=10\mathrm{ m}/\mathrm{s}$, respectively. Identification results of bridge damage with different speeds and different noise levels on the Class A road surface in the multi-damage case are shown in Fig. 13, the identification results with the speed of $15\mathrm{ m}/\mathrm{s}$ is shown in Fig. 12. The maximum relative errors of the damaged element stiffness under different vehicle speeds and noise levels are listed in Table 3.

**Table 3:** Damaged element stiffness identification errors with different speeds and different noises.

	*v* = 10 m/s			*v* = 15 m/s			*v* = 20 m/s		
	35 dB	30 dB	25 dB	35 dB	30 dB	25 dB	35 dB	30 dB	25 dB
${\alpha }_{3}$	0.27%	0.24%	0.34%	0.24%	0.31%	1.88%	0.59%	0.90%	1.89%
${\alpha }_{6}$	1.28%	0.56%	0.82%	1.46%	0.53%	0.43%	0.31%	1.13%	1.38%
${\alpha }_{9}$	0.09%	0.55%	1.09%	0.01%	0.81%	2.46%	1.54%	1.59%	2.47%

As can be seen in the Fig. 13 and Table 3, the identification results of the damage extent and the damage position are both very accurate. The maximum relative errors of the damaged element stiffness rise with the speed increase by the same noise.

The results mean that the influences of speed and noise level on the identification result are not significant. With the increase of the speed and noise level, all the damage identification results get less accurate. But the identification deviation of the damaged element stiffness does not exceed ± 0.05, even if the noise increases to 25 dB and speed rise to 20 m/s.

The results show that the maximum relative error of damage identification raises with the increase of the vehicle speed, because the amount of signal data measured reduces with the increase of the vehicle speed. Therefore, extremely fast vehicle speeds should be avoided in the damage detection, so that the measured data will be sufficient for damage parameters to be converged.

In this numerical example, the identified results are insensitive even if the speed is up to 20 m/s. The reason is that the amount of signal data measured reduced but still is enough to be identified accurately.

## Conclusions

In this work, an iterative approach for damage identification of the bridge structure based on extended Kalman filter (EKF) with $l1$-norm regularization algorithm is proposed. Both the road roughness and the parameters of the vehicle are unknown. In order to simplify the calculation process and improve the accuracy of the damage identification, the moving contact force is firstly identified, and then the bridge damage is identified. Numerical analysis of a simply supported bridge is carried out to verify the efficiency and accuracy of the proposed algorithm. The following conclusions can be obtained from the former observations and discussions:

1. The method is effective to identify damage in the bridge structures based on bridge vibration responses subject to moving contact loads. The introduction of $l1$-norm regularization makes it possible to obtain accurate identification results by suppressing noise interference.
2. The moving contact force is identified accurately without prior knowledge of the vehicular parameters on different vehicle speeds, damage cases, measurement noise levels, and roughness levels. The accurate identification of the contact moving contact force lays a solid foundation for the damage identification.
3. The bridge damage is insensitive to vehicle speeds, damage cases, measurement noise levels, and roughness levels. The ill-posedness of bridge damage identification is stronger compared with the moving contact force identification. The $l1$-norm regularization is necessary for the bridge damage identification.

There are many branches for Kalman filter method. The proposed method can be combined with nonlinear Kalman filters, adaptive Kalman filters and robust Kalman filters to improve the accuracy of the damage identification. Machine learning is also a popular approach to identify bridge cracks. Relevant work will be presented in the future.

## Supplementary Information

Supplementary Information.

## Data Availability

All data generated or analyzed during this study are included in this published article.
