# Supplementary material for: Extended Kalman filter algorithm for non-roughness and moving damage identification
Source: Sci Rep. 2022 Dec 19;12:21958. doi: 10.1038/s41598-022-26339-z (PMC9763337; doi:10.1038/s41598-022-26339-z)
Supplement: Supplementary file 1 — Supplementary Information. [file 41598_2022_26339_MOESM1_ESM.docx]

**Appendix 1**

$\boldsymbol{A}_{g}=\left[ \frac{\partial\boldsymbol{g}_{f}\left( \boldsymbol{\theta},t \right)}{\partial\boldsymbol{\theta}} \right]=\left[ \frac{\partial\boldsymbol{g}_{f}\left( \boldsymbol{\theta},t \right)}{\partial\boldsymbol{p}\left( t \right)} \frac{\partial\boldsymbol{g}_{f}\left( \boldsymbol{\theta},t \right)}{\partial\dot{\boldsymbol{p}}\left( t \right)} \frac{\partial\boldsymbol{g}_{f}\left( \boldsymbol{\theta},t \right)}{\partial f(\boldsymbol{x}^{*},t)} \right]$ (44a)

where

$\frac{\partial\boldsymbol{g}_{f}\left( \boldsymbol{\theta},t \right)}{\partial\boldsymbol{p}\left( t \right)}=\left[ \begin{matrix} \mathbf{0}_{1\times N} & -\boldsymbol{\Lambda} & \mathbf{0}_{1\times M} \end{matrix} \right]^{T}$ (45a)

$\frac{\partial\boldsymbol{g}_{f}\left( \boldsymbol{\theta},t \right)}{\partial\dot{\boldsymbol{p}}\left( t \right)}=\left[ \begin{matrix} \boldsymbol{I} & -\boldsymbol{\Gamma} & \mathbf{0}_{1\times M} \end{matrix} \right]^{T}$ (46a)

$\frac{\partial\boldsymbol{g}_{f}\left( \boldsymbol{\theta},t \right)}{\partial f(x,t)}=\left[ \begin{matrix} \mathbf{0}_{1\times N} & \boldsymbol{\Phi}\left( vt \right) & \mathbf{0}_{1\times M} \end{matrix} \right]^{T}$ (47a)

**Appendix 2**

Jacobian matrix：

$\boldsymbol{H}_{fi}\left( k+1 \right)=\left[ \frac{\partial\boldsymbol{h}_{f}\left( \boldsymbol{\theta}_{i}\left( k+1 \right),\boldsymbol{x}_{i}^{*},t_{k+1} \right)}{\partial\boldsymbol{\theta}_{i}\left( k+1 \right)} \right]={\left[ \begin{matrix} \begin{matrix} \frac{\partial\boldsymbol{h}_{f}}{\partial\boldsymbol{p}} & \frac{\partial\boldsymbol{h}_{f}}{\partial\dot{\boldsymbol{p}}} \end{matrix} & \frac{\partial\boldsymbol{h}_{f}}{\partial f} \end{matrix} \right]|}_{\theta=\theta_{k}}=$

$=\left\{ \begin{matrix} \left[ \begin{matrix} \left[ \begin{matrix} \boldsymbol{\varphi} & 0 \end{matrix} \right] & 0 & \left[ \begin{matrix} \frac{\partial\boldsymbol{\varphi}}{\partial f}\boldsymbol{p} & 0 \end{matrix} \right] \end{matrix} \right] & \mathrm{if}\boldsymbol{y}=\boldsymbol{\varphi p}\left( t \right) \\ \left[ \begin{matrix} 0 & \left[ \begin{matrix} \boldsymbol{\varphi} & 0 \end{matrix} \right] & \left[ \begin{matrix} \frac{\partial\boldsymbol{\varphi}}{\partial f}\dot{\boldsymbol{p}} & 0 \end{matrix} \right] \end{matrix} \right] & \mathrm{if}\boldsymbol{y}=\boldsymbol{\varphi}\dot{\boldsymbol{p}}\left( t \right) \\ \left[ \begin{matrix} \left[ \begin{matrix} -\boldsymbol{\varphi\Lambda} & 0 \end{matrix} \right] & \left[ \begin{matrix} -\boldsymbol{\varphi\Gamma} & 0 \end{matrix} \right] & \boldsymbol{\Phi}\left( vt \right) \end{matrix} \right] & \mathrm{if}\boldsymbol{y}=\boldsymbol{\varphi}\ddot{\boldsymbol{p}}\left( t \right) \end{matrix} \right.$ (48a)

**Appendix 3**

$\boldsymbol{A}_{B}=\left[ \frac{\partial\boldsymbol{g}_{\alpha}\left( \boldsymbol{\theta},t \right)}{\partial\boldsymbol{\theta}} \right]=\left[ \frac{\partial\boldsymbol{g}_{\alpha}\left( \boldsymbol{\theta},t \right)}{\partial\boldsymbol{p}\left( t \right)} \frac{\partial\boldsymbol{g}_{\alpha}\left( \boldsymbol{\theta},t \right)}{\partial\dot{\boldsymbol{p}}\left( t \right)} \frac{\partial\boldsymbol{g}_{\alpha}\left( \boldsymbol{\theta},t \right)}{\partial\boldsymbol{\alpha}} \right]$ (49a)

where：

$\frac{\partial\boldsymbol{g}_{\alpha}\left( \boldsymbol{\theta},t \right)}{\partial\boldsymbol{p}\left( t \right)}=\left[ \begin{matrix} \mathbf{0}_{1\times N} & -\boldsymbol{\Lambda} & \mathbf{0}_{1\times M} \end{matrix} \right]^{T}$ (50a)

$\frac{\partial\boldsymbol{g}_{\alpha}\left( \boldsymbol{\theta},t \right)}{\partial\dot{\boldsymbol{p}}\left( t \right)}=\left[ \begin{matrix} \boldsymbol{I} & -\boldsymbol{\Gamma} & \boldsymbol{0}_{1\times M} \end{matrix} \right]^{T}$ (51a)

$\frac{\partial\boldsymbol{g}_{\alpha}\left( \boldsymbol{\theta},t \right)}{\partial\boldsymbol{\alpha}}=\left[ \begin{matrix} \mathbf{0}_{1\times N} & -\frac{\partial\boldsymbol{\Gamma}}{\partial\alpha_{1}}\dot{\boldsymbol{p}}\left( t \right)-\frac{\partial\boldsymbol{\Lambda}}{\partial\alpha_{1}}\boldsymbol{p}\left( t \right)+\left( \frac{\partial\boldsymbol{\Phi}\left( vt \right)}{\partial\alpha_{1}} \right)^{T}\boldsymbol{f}(vt,t) & \mathbf{0}_{1\times M} & 0 \\ \vdots& \vdots& \vdots& \vdots\\ \mathbf{0}_{1\times N} & -\frac{\partial\boldsymbol{\Gamma}}{\partial\alpha_{M}}\dot{\boldsymbol{p}}\left( t \right)-\frac{\partial\boldsymbol{\Lambda}}{\partial\alpha_{M}}\boldsymbol{p}\left( t \right)+\left( \frac{\partial\boldsymbol{\Phi}\left( vt \right)}{\partial\alpha_{M}} \right)^{T}\boldsymbol{f}(vt,t) & \mathbf{0}_{1\times M} & 0 \end{matrix} \right]^{T}$ (52a)

where

$\frac{\partial\boldsymbol{\Gamma}}{\partial\alpha_{j}}=2\xi_{i}\frac{\partial\omega_{i}}{\partial\alpha_{j}}=2\xi_{i}\frac{\partial{\omega_{i}}^{2}}{\partial\alpha_{j}}\frac{1}{2\omega_{i}}=\xi_{i}\boldsymbol{\Lambda}^{-\frac{1}{2}}\frac{\partial\boldsymbol{\Lambda}}{\partial\alpha_{j}}$ (53a)

$\frac{\partial{\omega_{i}}^{2}}{\partial\alpha_{j}}\mathbf{=-}\boldsymbol{\varphi}_{i}^{T}\frac{\partial\boldsymbol{K}}{\partial\alpha_{j}}\boldsymbol{\varphi}_{i}$ (54a)

$\frac{\partial\boldsymbol{\Lambda}}{\partial\alpha_{j}}=diag\left( \frac{\partial{\omega_{1}}^{2}}{\partial\alpha_{j}} \frac{\partial{\omega_{2}}^{2}}{\partial\alpha_{j}} \cdots\frac{\partial{\omega_{N}}^{2}}{\partial\alpha_{j}} \right)=2\left[ \begin{matrix} \omega_{1} & \cdots& 0 \\ \vdots& \ddots& \vdots\\ 0 & \cdots& \omega_{N} \end{matrix} \right]\left[ \begin{matrix} \frac{\partial\omega_{1}}{\partial\alpha_{j}} & \cdots& 0 \\ \vdots& \ddots& \vdots\\ 0 & \cdots& \frac{\partial\omega_{N}}{\partial\alpha_{j}} \end{matrix} \right]$ (55a)

sensitivity matrix of the corresponding feature matrix:

$\frac{\partial\boldsymbol{\Phi}\left( vt \right)}{\partial\alpha_{j}}\mathbf{=}\left[ \frac{\partial\boldsymbol{\varphi}_{1}\left( vt \right)}{\partial\alpha_{j}} \frac{\partial\boldsymbol{\varphi}_{2}\left( vt \right)}{\partial\alpha_{j}}\boldsymbol{\cdots}\frac{\partial\boldsymbol{\varphi}_{N}\left( vt \right)}{\partial\alpha_{j}} \right]$ (56a)

$\frac{\partial\boldsymbol{\varphi}_{N}\left( vt \right)}{\partial\alpha_{j}}\mathbf{=}\sum_{k=1,k\neq r} \frac{\boldsymbol{\varphi}_{k}^{T}\boldsymbol{K}\boldsymbol{\varphi}_{r}^{T}}{\left( {\omega_{k}}^{2}-{\omega_{r}}^{2} \right)}\boldsymbol{\varphi}_{k}$ (57a)

**Appendix 4**

Jacobian matrix：

$\boldsymbol{H}_{\alpha i}\left( k+1 \right)=\left[ \frac{\partial\boldsymbol{h}_{\alpha}\left( \boldsymbol{\theta}_{i}\left( k+1 \right),\boldsymbol{x}_{i}^{*},\boldsymbol{t}_{k+1} \right)}{\partial\boldsymbol{\theta}_{i}\left( k+1 \right)} \right]={\left[ \begin{matrix} \begin{matrix} \frac{\partial\boldsymbol{h}_{\alpha}}{\partial\boldsymbol{p}} & \frac{\partial\boldsymbol{h}_{\alpha}}{\partial\dot{\boldsymbol{p}}} \end{matrix} & \frac{\partial\boldsymbol{h}_{\alpha}}{\partial\boldsymbol{\alpha}} \end{matrix} \right]|}_{\theta=\theta_{k}}=\left\{ \begin{matrix} \left[ \begin{matrix} \left[ \begin{matrix} \boldsymbol{\varphi} & 0 \end{matrix} \right] & 0 & \left[ \begin{matrix} \frac{\partial\boldsymbol{\varphi}}{\partial\boldsymbol{\alpha}}\boldsymbol{p} & 0 \end{matrix} \right] \end{matrix} \right] & \mathrm{if}\boldsymbol{y}=\boldsymbol{\varphi p}\left( t \right) \\ \left[ \begin{matrix} 0 & \left[ \begin{matrix} \boldsymbol{\varphi} & 0 \end{matrix} \right] & \left[ \begin{matrix} \frac{\partial\boldsymbol{\varphi}}{\partial\boldsymbol{\alpha}}\dot{\boldsymbol{p}} & 0 \end{matrix} \right] \end{matrix} \right] & \mathrm{if}\boldsymbol{y}=\boldsymbol{\varphi}\dot{\boldsymbol{p}}\left( t \right) \\ \left[ \begin{matrix} \left[ \begin{matrix} \boldsymbol{-\varphi\Lambda} & 0 \end{matrix} \right] & \left[ \begin{matrix} \boldsymbol{-\varphi\Gamma} & 0 \end{matrix} \right] & \tilde{\boldsymbol{H}} \end{matrix} \right] & \mathrm{if}\boldsymbol{y}=\boldsymbol{\varphi}\ddot{\boldsymbol{p}}\left( t \right) \end{matrix} \right.$ (58a)

where

$\tilde{\boldsymbol{H}}=\begin{matrix} \frac{\partial\boldsymbol{\varphi}}{\partial\boldsymbol{\alpha}}\ddot{\boldsymbol{p}}+\boldsymbol{\varphi}\left( -\frac{\partial\boldsymbol{\Gamma}}{\partial\boldsymbol{\alpha}}\dot{\boldsymbol{p}}\left( t \right)-\frac{\partial\boldsymbol{\Lambda}}{\partial\boldsymbol{\alpha}}\boldsymbol{p}\left( t \right)+\left( \frac{\partial\boldsymbol{\Phi}\left( vt \right)}{\partial\boldsymbol{\alpha}} \right)^{\boldsymbol{T}}\boldsymbol{f}\left( vt,t \right) \right) \end{matrix}$ (59a)
